# Low prevalence of ‘classical’ microscopic colitis but evidence of microscopic inflammation in Asian Irritable Bowel Syndrome patients with diarrhoea

**DOI:** 10.1186/1471-230X-13-80

**Published:** 2013-05-08

**Authors:** Ida Hilmi, Juanda Leo Hartono, Jayalakshmi Pailoor, Sanjiv Mahadeva, Khean-Lee Goh

**Affiliations:** 1 Gastroenterology Unit, Department of Medicine, Faculty of Medicine, University of Malaya, Kuala Lumpur, Malaysia; 2 Department of Pathology, Faculty of Medicine, University of Malaya, Kuala Lumpur, Malaysia

## Abstract

**Background:**

There is increasing evidence for the role of microscopic inflammation in patients with IBS. We aimed to examine the prevalence of microscopic colitis and inflammation in Malaysian IBS patients with diarrhoea (IBS-D).

**Methods:**

Consecutive patients who met the Rome III criteria for IBS-D and asymptomatic controls were prospectively recruited. Colonoscopy was performed in all study subjects and systematic biopsies taken from all segments of the colon. The diagnosis of lymphocytic colitis and collagenous colitis was made using previously defined criteria. Patients with post infectious IBS were excluded.

**Results:**

120 subjects (74 IBS-D, 46 controls) were recruited during the study period. In the IBS-D group, the colonoscopic (macroscopic) findings were as follows; normal findings n = 58 (78.4%), diverticula disease n = 5 (6.8%), diminutive polyps n = 9 (12.2%) and haemorrhoids n = 2(2.7%). No subject under the age of 40 had any significant findings. Microscopically, there was only one case (1.3%) with histology consistent with collagenous colitis. However, the IBS-D patients had a higher prevalence of moderate microscopic inflammation (n = 11, 14.9%) compared to controls (n = 1, 2.2%) (p = 0.005).

**Conclusions:**

‘Classical’ microscopic colitis is uncommon in Malaysian patients with IBS-D but a significant number of adults showed evidence of microscopic inflammation.

## Background

Irritable Bowel Syndrome (IBS) is defined as a chronic abdominal discomfort associated with altered bowel habit. It is a common gastrointestinal disorder worldwide, with prevalence rates ranging from 2-22% in the West (depending on criteria used) [1] and a rate of 15% in the Malaysian population, based on the Rome II criteria [2,3]. The Rome criteria, the most recent modification being the Rome III criteria [4], allows a positive diagnosis of IBS based on symptoms alone and additionally allows for subtype classification depending on the predominant symptom. The exact pathogenesis of IBS is generally unknown but postulated mechanisms include alterations in gut motility, visceral hypersensitivity, bacterial flora, together with psychological association [5-7]. Recently, there has been an increased interest in the role of microscopic inflammation in patients with IBS [7-14].

‘Classical’ microscopic colitis is a histopathological diagnosis which is currently accepted as an umbrella term for either lymphocytic colitis or collagenous colitis. Both subtypes share similar histological features, apart from the fact that collagenous colitis has a significantly thickened sub-epithelial collagen layer. The annual incidence in western countries ranges from 1 to 12 per 100,000 and is a common finding (10-15%) in patients investigated for chronic diarrhoea [15]. It is a disease of the elderly with a strong female preponderance. The pathogenesis is not fully elucidated but autoimmunity, luminal antigens, drugs such as NSAIDs and bile salt malabsorption have been implicated [15]. The clinical symptoms include chronic diarrhoea, urgency, incontinence, anorexia, nausea, abdominal cramping, and mild weight loss. Not surprisingly, a significant number of patients who have microscopic colitis also fulfill the Rome criteria for IBS [16-18]. Therefore, a subset of IBS patients, in particular those with diarrhoea (IBS-D) who do not undergo colonoscopy and biopsies would have missed a diagnosis of microscopic colitis. This is clinically relevant as there is effective treatment, in particular budesonide, which has been specifically licensed for this condition [15,19-22]. In addition, a diagnosis of microscopic colitis should prompt a search for associated autoimmune conditions such as coeliac disease.

As IBS represents a significant health problem in Malaysia, the aim of this study was to examine the prevalence of microscopic colitis in Malaysian patients with IBS-D. In view of the increasing evidence for the role of inflammation in patients with IBS, our secondary objective was to examine the histopathological features in these patients compared to controls.

## Methods

This study was approved by the University of Malaya Medical Centre (UMMC) Ethics Committee. Consecutive patients who presented to the UMMC Endoscopy Unit, who met the Rome III criteria for IBS-D from May 2010 to May 2011, were prospectively recruited following informed consent. These patients were primarily referred from primary care physicians for further investigation, and none had undergone prior colonoscopy. In our clinical practice, a colonoscopy is usually requested after a negative screen for organic causes of diarrhoea such as parasitic infestation, thyroid disease, etc. However, screening for Coeliac Disease and Tropical Sprue are not routinely performed as they are rare in our population. The controls chosen during the same time period were asymptomatic subjects who had undergone colonoscopy for colorectal cancer screening or polyp surveillance. Subjects with the following features were excluded: prior gastrointestinal surgery, significant loss of weight, bloody stool, metabolic disorders such as thyrotoxicosis, corticosteroid usage in the past 4 weeks and those with a recent GI infection (i.e. indicative of post-infectious IBS).

Baseline demographic characteristics, detailed GI symptoms, smoking history, alcohol intake, drug history and concomitant medical illness were documented. A single stool sample from each patient was sent for microscopy and culture.

### Colonoscopy and biopsy

For all subjects, the bowel preparation used was polyethylene glycol (Fortrans®). During colonoscopy, the macroscopic findings were recorded. Two well-oriented biopsies were taken from each part of the colon (i.e. caecum, ascending colon, transverse colon, descending colon, sigmoid, and rectum) which appeared macroscopically normal. The biopsies were fixed in 10% buffered formalin and sent for processing. The tissues were routinely processed for light microscopic examination. The paraffin embedded biopsy materials were sectioned and stained with the Hematoxylin and eosin stain. Additional stains such as Masson trichrome stains for collagen fibres were done when collagenous colitis was suspected on H&E stains.

### Histopathological assessment

Biopsy specimens were assessed by an experienced pathologist who was blinded to the clinical indication for colonoscopy. Quantitative assessment of intraepithelial lymphocytes, intraepithelial neutrophils, subepithelial collagen thickness, and lamina propria infiltration (by lymphocytes, plasma cells, and mononuclear cells) were documented as well as the presence of crypt distortion and surface epithelial damage. The histological criteria for the diagnosis of lymphocytic colitis was as follows; increased intraepithelial lymphocytes (IEL) of 20 or more per 100 epithelial cell in conjunction with surface epithelial damage, normal collagen layer and normal crypt architecture. The histological criteria for collagenous colitis was abnormally thickened subepithelial collagen band of 10 μm or more, chronic inflammation including increased IEL and normal crypt architecture [23].

## Results

### Demography

One hundred and twenty subjects, 74 who fulfilled the Rome III criteria and 46 controls were recruited. The demography of both cases (IBS-D patients) and controls are highlighted in Table 1. Among IBS-D patients, the male:female ratio was 1:1.4, and the median age was 51 years. The median stool frequency was 4 times per day (range 2–10), the median duration of symptoms was 12 months and nocturnal diarrhoea was seen in 3 (4%) subjects. In the control group, the male:female ratio was 1:0.9, and the median age was 62 years.

**Table 1:** Summary of baseline characteristics, endoscopic and histological findings in patients with IBS-D and controls

**IBS-D n(%)**		**Controls n(%)**	
**Total**	74	**Total**	46
**Gender**		**Gender**	
Male	31(41.9%)	Male	24(52.2%)
Female	43(58.1%)	Female	22(47.8%)
**Median age(years)**	51(16–78)	**Median(range)**	61(26–79)
**Race**		**Race**	
Malay	15(20.3%)	Malay	8(17.4%)
Chinese	36(48.6%)	Chinese	31(66%)
Indian	23(31.1%)	Indian	7(14.9%)
**Colonoscopic findings**		**Colonoscopic findings**	
Normal	58 (78.4%)	Normal	27(58.7%)
Polyps	9 (12.2%)	Polyps	15(32.6%)
Diverticular disease	5 (6.8%)	Diverticular disease	3(6.5%)
Haemorrhoids	2(2.7%)	Haemorrhoids	1(2.2%)
**Histological findings**		**Histological findings**	
Normal	62(83.8%)	Normal	0(97.8%)
Collagenous	1(1.4%)	Collagenous colitis	0(0)
Lymphocytic colitis	0(0%)	Lymphocytic colitis	0(0)
Non specific colitis	11(14.9%)	Non specific colitis	1(2.2%)

### Colonoscopy findings

The colonoscopy (macroscopic) findings in the IBS-D group were as follows; normal findings n = 58 (78.4%), diverticula disease n = 5 (6.8%), diminutive polyps n = 9 (12.2%) and haemorrhoids n = 2(2.7%). No subject under the age of 40 had any significant findings. Colonoscopy findings in the control group were as follows: normal findings n = 27(58.7%), adenomas n = 15(32.6%) (one large rectal polyp 1 cm, the others <1 cm), diverticula disease n = 3(6.5%) and haemorrhoids n = 1(2.2%).

### Histological findings

**Figure 1:**
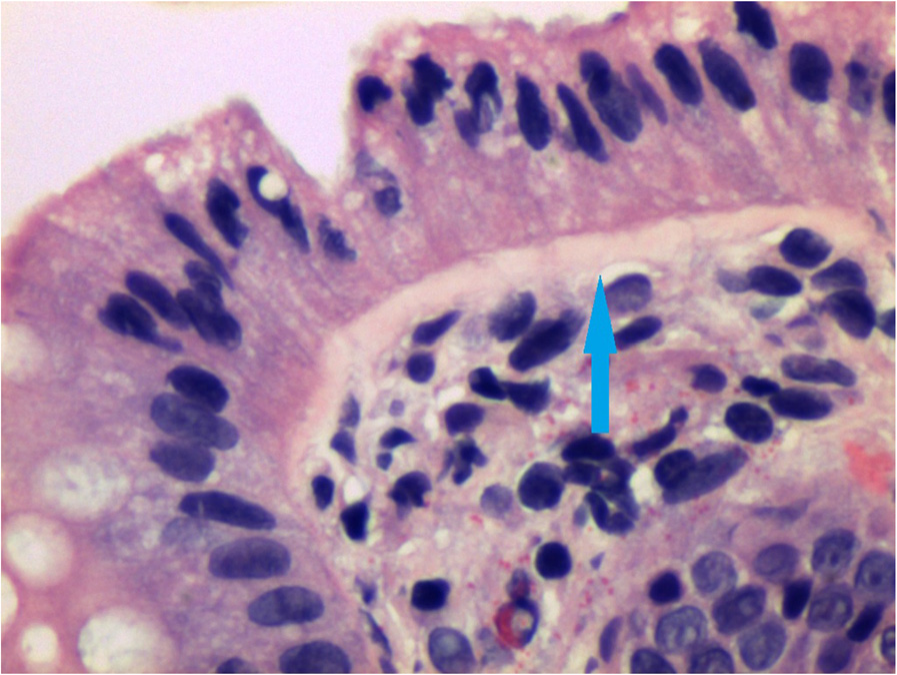
**IBS-D patient with collagenous colitis.** Note the thickened collagen band (arrow).

Collagenous colitis was diagnosed in a single, 63-year old female IBS-D patient, of Indian ethnicity. The histological features of thickened collagen fibres were observed in the transverse, descending, sigmoid colon and rectum (Figure 1). There were no subjects who fulfilled the criteria for lymphocytic colitis.

**Figure 2:**
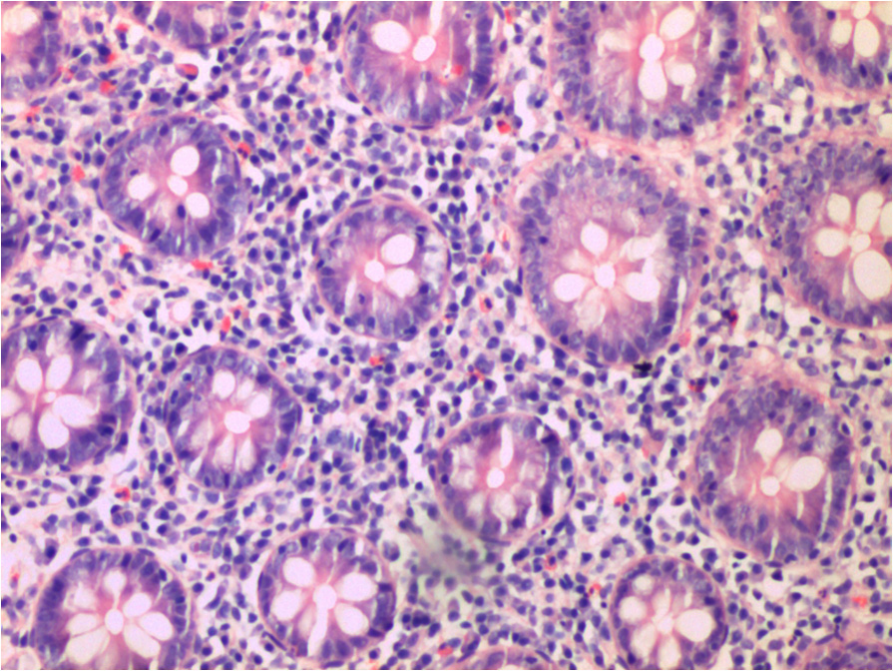
IBS -D patient with evidence of moderate to severe inflammation on histology.

**Figure 3:**
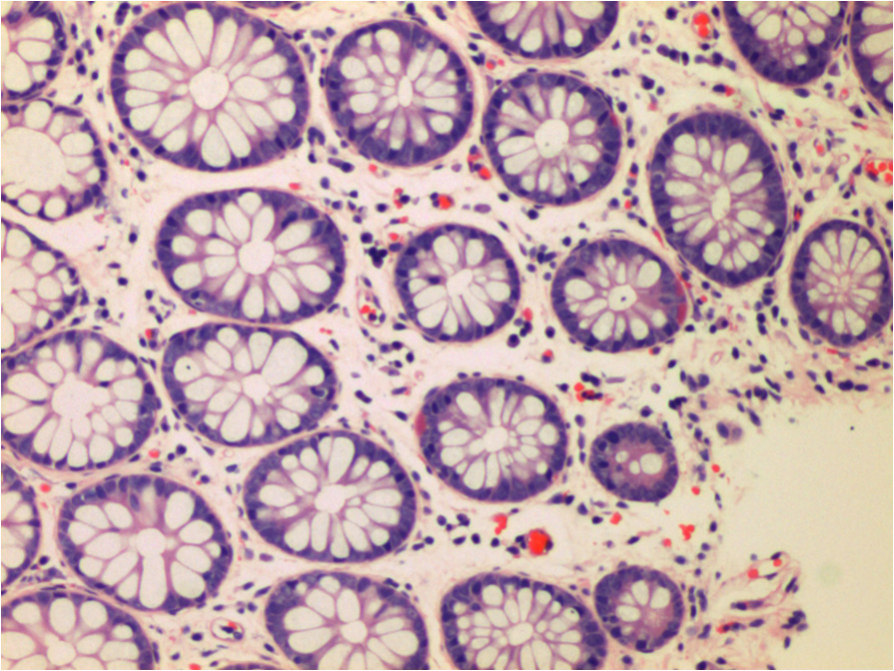
IBS-D patient with normal histology.

A further 11/74 (14.9%) IBS-D cases showed evidence of microscopic inflammation, with moderate lymphocytic and plasmacytic infiltration in the lamina propria. Eight cases had distal involvement but in four cases, inflammation was seen only proximal to the splenic flexure only. Infiltration of an occasional crypt by neutrophils was seen in five of the cases and scattered crypt abscesses in the transverse colon, sigmoid colon and rectum were present in one case. Moderate infiltration of the lamina propria only without crypt involvement was noted in the remaining five cases. An example of one of the cases is seen in Figure 2. The remaining 62 (83.7%) subjects with IBS-D did not show any evidence of significant inflammation throughout the colon (Figure 3).

Amongst those in the control group, moderate infiltration of lymphocytes in the ascending and transverse colon, with no evidence of cryptitis, was found in 1/46 (2.2%) adult. This patient was an asymptomatic, 60-year-old Chinese male, who had undergone a colonoscopy for colorectal cancer screening. None of the other subjects in the control group showed any evidence of significant microscopic inflammation. Univariate analysis demonstrated that IBS-D subjects, compared to controls, had a greater prevalence of moderate to severe microscopic inflammation (14.9% vs 2.2%, OR 11.44, 95% CI = 1.49-240.69, p = 0.005).

## Discussion

There are several relevant observations in our study. The primary objective was to look at the prevalence of microscopic colitis in patients who were diagnosed with IBS-D as defined by the Rome III criteria. From the study, it appears that classic microscopic colitis is very uncommon in our cohort of patients with IBS-D. However, a significant percentage of subjects with IBS-D had evidence of microscopic inflammation that did not fit the criteria for classical microscopic colitis. The most common abnormalities seen were mixed chronic and acute inflammatory cells, lymphocytes, plasma cells and neutrophils; with or without cryptitis and crypt abscesses.

The categories of microscopic colitis were recently expanded by Falodia et al. into five subtypes: collagenous colitis, lymphocytic colitis, minimal change colitis (crypt architectural abnormality in the form of cryptitis and crypt dilatation in the absence of increase in intraepithelial lymphocytes and subepithelial collagenous band), microscopic colitis not otherwise specified (increased inflammatory cell infiltrates in the lamina propria in the absence of other abnormalities) and microscopic colitis with giant cells [24]. If we were to reclassify our patients according to the above categories, one patient had collagenous colitis, six patients had minimal change colitis and five patients had microsopic colitis not otherwise specified. However, the classification proposed by Falodia et al. has yet to be widely accepted and the therapeutic studies carried out in the past only looked specifically at collagenous and lymphocytic colitis. Inflammation was patchy throughout the colon and from the study, four cases showed inflammation beyond the splenic flexure. This is similar to classical microscopic colitis, where the diagnosis can be missed unless a complete colonoscopy with biopsies throughout the colon is conducted [15].

The role of inflammation in IBS has been of interest in recent times and evidence of low level inflammation has been demonstrated in both post infectious and non-post infectious IBS. Gwee et al. found increased expression of pro-inflammatory cytokine interleukin Iβ (IL-1β) mRNA in subjects with post-infectious IBS, which was not observed in adults without IBS [12]. Chadwick et al. [25,26] had divided the histological findings of IBS patients into three groups; those with normal histology, those with evidence of microscopic inflammation and those who met criteria for lymphocytic colitis. With immunostaining, it was found that all three groups had increased numbers of activated immunocompetent cells in the intestinal mucosa. Barbara et al. [27] reported that increased numbers of activated mast cells in close proximity to intestinal innervation correlated with abdominal pain in IBS. In addition to this, there is evidence that patients with IBS-D have an increased expression of TNFα and IL-1β in the peripheral blood monocytes, not dissimilar to that found in patients with inflammatory bowel disease [8]. A study from Sri Lanka also showed low grade inflammation in IBS patients, similar to Western studies but with an increase in eosinophils as well as the other chronic inflammatory cells [28].

An attractive concept is that IBS-D forms the mildest part of a disease spectrum, with idiopathic inflammatory bowel disease (in particular, ulcerative colitis) at the other end, with classical microscopic colitis in between [29,30]. There is a clear overlap in terms of their proposed pathogenesis including alterations in intestinal microbiota and bile salt malabsorption. However, the common pathways between the conditions remain poorly understood and the majority of IBS-D patients fail to demonstrate significant inflammation on histology.

In the light of Western studies demonstrating a significant overlap between classical microscopic colitis and IBS-D, as well as the increasing role of inflammation in IBS patients, ‘to do or not to do’ colonoscopy in patients with IBS-D remains as unclear as ever. Although the single case of collagenous colitis in our study was found in the ‘typical’ subject (elderly, female), non specific inflammation was seen in both age groups, both genders and across all three races. As the prevalence of collagenous and lymphocytic colitis appears to be low in our population, routine colonoscopy is not justified in young patients who have been identified as IBS on symptom based criteria. This may change however, in light of promising data on the use of anti-inflammatory drugs such as mesalazine in IBS [31-34]. It is uncertain, however, if only those with evidence of microscopic inflammation will respond to anti-inflammatory therapy, thereby necessitating the use of routine colonoscopy in order to provide a more individualised approach to the management of these patients.

The low colonoscopic yield among IBS patients in this study is noteworthy. No macroscopic abnormalities were seen in subjects under the age of forty and it is also reassuring to note that no significant pathology was identified in the older adults. In fact, our findings were very similar to a large study carried out in USA where findings of polyps, diverticular disease and haemorrhoids in subjects with IBS were no different to that of asymptomatic controls [35]. While it is certainly reasonable to still offer colonoscopy in older patients with IBS for colorectal cancer screening, the study emphasizes the importance of careful history taking in stratifying patients who do or do not require colonoscopy and those who require it urgently or otherwise. This is especially relevant in Malaysia, which is still considered a developing country, with limited endoscopic facilities and only 106 registered gastroenterologists in a population of 25 million [36].

There are several limitations of the study. The study sample was small and patients were derived from secondary care, which may not be representative of IBS patients in the community. As IBS, like other functional GI diseases, is rarely life-threatening, medical consultation rates amongst sufferers are known to be low. CD3 staining for lymphocytes was not carried out which may have resulted in an under-diagnosis of lymphocytic colitis [37]. Screening for Coeliac Disease, rare among our population but known to be associated with collagenous colitis, was not performed. However, the merits of this study lay in our strict adherence to the Rome III criteria and systematic sampling of colonic mucosal tissue, providing an accurate estimation of inflammation in an Asian population with IBS-D.

## Conclusions

In this prospectively conducted study, we have found a significant proportion of patients with IBS-D who have evidence of colonic inflammation on biopsies which may be part of a microscopic colitis spectrum. This further adds to the growing evidence for the role of inflammation which will hopefully result in increasing the therapeutic options for the management of this common but difficult to treat condition.

## Competing interests

All authors declare no competing interests in the conduct of this study and preparation of the manuscript.

## Authors’ contributions

IH - research design, drafting of manuscript; JLH - research design, acquisition of data; JP - analysis and interpretation of results; SM - research design, acquisition of data, critical review of manuscript; GKL - critical review of manuscript. All authors read and approved the final manuscript.

## Pre-publication history

The pre-publication history for this paper can be accessed here:

http://www.biomedcentral.com/1471-230X/13/80/prepub
